# Analysis of screen-detected pulmonary nodules before and after the novel coronavirus epidemic: a multicenter retrospective cohort study

**DOI:** 10.3389/fonc.2025.1534074

**Published:** 2025-04-01

**Authors:** Zemin He, Keting Liu, Ling Wu, Qiang Wei, Qingwei Shen

**Affiliations:** ^1^ Department of Thoracic Surgery, The First People’s Hospital of Shuangliu District (West China Airport Hospital of Sichuan University), Chengdu, Sichuan, China; ^2^ Department of Neurology, Chengdu Seventh People’s Hospital, Chengdu, Sichuan, China; ^3^ Department of Respiratory Medicine, The First People’s Hospital of Shuangliu District (West China Airport Hospital of Sichuan University), Chengdu, Sichuan, China; ^4^ Department of Thoracic Surgery, Sichuan Baoshi Flower Hospital, Chengdu, Sichuan, China

**Keywords:** chest CT, lung cancer screening, lung nodules, COVID-19 epidemic, review

## Abstract

**Objective:**

To analyze the screening results for pulmonary nodules before and after the COVID-19 epidemic to understand the influence of the COVID-19 epidemic on the detection rate of pulmonary nodules and the detection rate of high-risk pulmonary nodules.

**Methods:**

A total of 18,906 chest CT scans were performed between March and November 2022 and between March and November 2023. Subjects from March to December 2022 were divided into the pre-epidemic group, and subjects from March to December 2023 were divided into the post-epidemic group. The detection rates of lung nodules, high risk nodules, different age groups, and gender groups were analyzed.

**Results:**

A total of 11513 lung nodules were detected during screening. A total of 841 high risk nodules were detected, and the detection rate of solid nodules was significantly higher in the postepidemic group than in the pre-epidemic group. The detection rate of fibrotic changes was significantly higher in the postepidemic group than in the pre-epidemic group. The detection rate of pulmonary nodules is significantly higher in those aged > 51 years compared to those aged 50 years and below.

**Conclusion:**

In the post-COVID-19 period, there has been a significant increase in the detection of solid pulmonary nodules. This increased detection rate is predominantly observed in medical patients over 51 years of age. However, the COVID-19 epidemic has not resulted in an increased detection rate of high risk nodules.

## Introduction

The mortality rate and incidence of lung cancer are both notably high. Early screening and diagnosis are critical to the improvement of patient survival rates (1). Since December 2019, cases of novel coronavirus disease (COVID-19) caused by severe acute respiratory syndrome coronavirus 2 (SARS-CoV-2) have been reported in many places in China. The virus has spread rapidly among humans, causing COVID-19 pneumonia. Initially, the novel coronavirus was effectively contained due to the stringent quarantine measures implemented by the Chinese government (2). But as the epidemic unfolded, Chengdu, China, was eventually forced to lift quarantine restrictions on novel coronaviruses in December 2022.The rapid propagation of the virus has had a substantial impact on the screening, diagnosis, and treatment of pulmonary nodules.

Previous studies have shown that some patients with COVID-19 have CT imaging characteristics similar to those of patients with early-stage lung cancer, showing solid or ground-glass nodules, which undoubtedly increases the difficulty of differential diagnosis and treatment (3, 4). There are also studies suggesting that the inflammatory response induced by COVID-19 may lead to elevated levels of inflammatory markers such as IL-6, which play a key role in angiogenesis and metastasis and may accelerate the development of lung cancer (5). Some studies (6) have also shown that the post-COVID-19 period led to an increase in the number of lung nodules detected. However, these studies found that the increase in the detection rate of lung nodules was due to an increase in the number of detections rather than an increase in the incidence rate of lung nodules themselves. Other studies (7) have shown that lung cancer patients often have a variety of complications, such as obstructive pneumonia, lung infection, radiation pneumonia and pneumonia after immunotherapy. The differential diagnosis between lung cancer and COVID-19 is complicated by the presence of these complications. However, most of the studies were single-center studies with small sample sizes, and little attention was paid to the detection rates of different types of lung nodules and high risk nodules before and after COVID-19. To date, the detection rate of nodules and lung cancer after COVID-19 is unclear.

Therefore, we conducted a multicenter retrospective cohort study with a focus on a comprehensive analysis of changes in detection rates of lung nodules and high-risk nodules before and after the COVID-19 epidemic. By comparing screening data from three hospitals, we aim to clarify the specific impact of COVID-19 on the detection rate of different types of lung nodules, especially in different age and gender groups. This study will not only help to understand the potential relationship between COVID-19 and lung nodules, but will also provide an important reference for optimizing lung cancer screening strategies and post-epidemic public health policies.

## Data and methods

### Subjects investigated

The study subjects were 18906 who completed chest CT examinations at Chengdu Shuangliu District First People’s Hospital, Chengdu Seventh People’s Hospital, and Sichuan Baoshihua Hospital from March to November 2022 and March to November 2023. The inclusion criteria for the study subjects were as follows: a) all study subjects settled in the Chengdu area; b) no obvious symptoms of upper respiratory tract infection, such as cough and sputum; and c) voluntarily completed chest CT. The exclusion criteria were as follows: a) pregnancy or preparation for pregnancy; b) history of malignancy; c) tuberculosis or severe pulmonary infection; and d) obvious chest pain, cough, sputum, or hemoptysis during physical examination. It is important to note that the above exclusion criteria were applied during the retrospective cohort analysis and were not part of the original lung cancer screening protocol. The novel coronavirus quarantine in Chengdu, China, was lifted in December 2022, and the pandemic lasted for approximately two months. Therefore, we set the study subjects from March to November 2022 as the preepidemic group and those from March to November 2023 as the postepidemic group. As our research subjects were sourced from health screening center data, some individuals under the age of 40 were also included in our study. Their reasons for undergoing health screening were mainly due to the company’s enhanced health screening package or improving personal health management awareness, rather than clinical indications.

### Inspection method

Using spiral CT of 64 layers and above, a thin CT scan was performed for all patients. The examination position was as follows: supine position, with both arms placed above the head; the scan was completed at the end of inspiration, ranging from the tip of both lungs to the bilateral costal diaphragmatic angle. The scanning parameters were as follows: tube voltage, 120 kV; tube current, 40 mA; layer thickness, 2 mm; and layer distance, 1 mm. Image reconstruction was completed after scanning.

### The diagnosis of the report

Each CT report was reviewed by two senior radiology physicians via the imaging archiving and picture archiving and communication system (PACS). If there was disagreement, further discussion of the report was needed. The report should describe the details of the pulmonary nodules, including their location, size, number, density, and morphology. All pulmonary nodules were classified via the Lung Reporting and Data System (Lung-RADS) (8). These high-risk nodules were classified as Class 3 and Class 4 nodules by the Lung-RADS imaging system (9) and evaluated by two or more thoracic surgeons in a joint review. We define a nodule as high-risk only when two physicians agree that it is high-risk.

### Definition and classification of fibrotic lesions

In this study, fibrotic lesions were defined as chronic parenchymal abnormalities on chest CT scans characterized by reticular patterns, traction bronchiectasis, honeycombing, or linear scars. These were classified as nonspecific interstitial fibrosis, as opposed to nodular fibrosis or acute inflammatory changes. Classification followed the 2018 ATS/ERS guidelines (10) for idiopathic pulmonary fibrosis, and was adjusted to distinguish COVID-19-related fibrotic changes from pre-existing fibrosis. Two senior radiologists independently reviewed all cases, and disagreements were resolved by consensus.

### Statistical analysis

The data were analyzed via SPSS 19.0. The normally distributed data are expressed as the mean ± standard deviation (x ± s), and an independent samples t test was used for comparisons between groups. Count data are presented as cases (%), and the χ ^2^ test was used for comparisons between groups. Statistical significance was set at <0.05. To adjust for potential confounders, we performed multivariate logistic regression analysis, including variables with p values less than 0.10 from the univariate analysis.

## Results

### Basic information of the study subjects

Among the 18,906 participants, 11,757 (62.2%) were male and 7,149 (37.8%) were female. The cohort was divided into two groups: 8,824 participants in the pre-pandemic group and 10,082 in the post-pandemic group. The mean age of the post-pandemic group was lower than that of the pre-pandemic group, and the proportion of lung nodule screening varied across different age groups. Notably, the proportion of lung nodule screening increased in the age group ≤40 years in the post-pandemic period compared to the pre-pandemic period. Further multivariate analysis revealed statistically significant differences between the two groups in terms of age, sex, fibrosis, and solid nodules. Detailed information is presented in **Table 1**.

**Table 1:** Comparison of the preepidemic and postepidemic groups.

	Preepidemic group (n=8824)	Postepidemic group (n=10082)	χ2/t	P	Multivariate analysis	
					P	OR (95%CI)
Age (years) ± SD	48.28 ± 11.97	46.97 ± 12.53	7.372	<0.001	<0.001	0.975 (0.968~0.982)
Sex [n (%)]			9.156	0.002	0.010	1.082 (1.019~1.148)
Male	5588 (63.3)	6169 (61.2)				
Female	3236 (36.7)	3913 (38.8)				
Age (years) [n (%)]			49.869	<0.001	<0.001	1.220 (1.120~1.328)
≤40 (n=5843)	2506 (28.4) a	3337 (33.1) a				
41~50 (n=5063)	2450 (27.8) b	2613 (25.9) b				
51~60 (n=5685)	2771 (31.4) b	3914 (28.9) b				
≥61 (n=2315)	1097 (12.4) b	1218 (12.1) b				
pulmonary nodule [n (%)]			5.635	0.018	0.605	0.984 (0.925~1.047)
yes	5294 (60.0)	6219 (61.7)				
no	3530 (40.0)	3863 (38.3)				
high risk nodules [n (%)]			0.549	0.459		
yes	403 (4.6)	438 (4.3)				
no	8421 (95.4)	9644 (95.7)				
pGGN [n (%)]			3.768	0.052		
yes	871 (9.9)	1082 (10.7)				
no	7953 (90.1)	9000 (89.3)				
mGGN[n (%)]			3.446	0.063		
yes	27 (0.3)	48 (0.5)				
no	8797 (99.7)	10034 (99.5)				
solid nodules [n (%)]			41.056	<0.001	<0.001	0.738 (0.678~0.803)
yes	1090 (12.4)	1573 (15.6)				
no	7734 (87.6)	8509 (84.4)				
multiple nodules[n (%)]			0.790	0.374		
yes	1383 (15.7)	1533 (15.2)				
no	7441 (84.3)	8459 (84.8)				
fibrosis lesions [n (%)]			19.133	<0.001	<0.001	0.810 (0.761~0.863)
yes	2640 (29.9)	3315 (32.9)				
no	6184 (70.1)	6767 (67.1)				
calcifications [n (%)]			3.187	0.076		
yes	2825 (32.0)	3106 (30.8)				
no	5999 (68.0)	6976 (69.2)				
LungRads [n (%)]			6.75	0.080		
1	5060 (57.34)	5951 (59.03)				
2	2735 (31.00)	3035 (30.10)				
3	626 (7.09)	685 (6.79)				
4	403 (4.57)	411 (4.08)				

### Results of the detection of pulmonary nodules

A total of 11,513 pulmonary nodules were detected in all the study subjects, for a detection rate of 60.9%. The detection rate in the pre-epidemic group was lower than that in the post-epidemic group. Analysis of the different characteristics of pulmonary nodules showed that the detection rates of pure ground glass nodules (pGGNs), mixed ground glass nodules (mGGNs), multiple nodules, and calcifications in the pre-endemic group were not different from those in the post-endemic group. Among all pulmonary nodule subtypes, only solid nodules showed a statistically significant increase in detection rate during the post-pandemic period. The detection rate of pulmonary fibrotic lesions in the post-epidemic group was significantly higher than that in the pre-epidemic group. According to the analysis of different age groups, The detection rate of pulmonary nodules is significantly higher in those aged > 51 years compared to those aged 50 years and below. There was no statistically significant difference in the classification distribution of LungAds between the two groups (p>0.05).Details are shown in **Tables 1** and **2**.

**Table 2:** Comparison of the detection rates of pulmonary nodules at different ages between the preepidemic and postepidemic groups.

	Preepidemic group		Postepidemic group		χ2	P
	[n]	Pulmonary nodule [n (%)]	[n]	Pulmonary nodule [n (%)]		
Sex [n (%)]						
Male (n=11757)	5588	3344 (59.8)	6169	3799 (61.6)	3.721	0.054
Female (n=7149)	3236	1950 (60.3)	3913	2420 (61.8)	1.874	0.171
Age (years) [n (%)]						
≤40 (n=5843)	2506	1478 (59.0)	3337	2035 (61.0)	2.398	0.121
41~50 (n=5063)	2450	1474 (60.2)	2613	1546 (59.2)	0.523	0.470
51~60 (n=5685)	2771	1666 (60.1)	2914	1839 (63.1)	5.358	0.021
≥61 (n=2315)	1097	676 (61.6)	1218	799 (65.6)	3.948	0.047

### Results of the detection of high risk nodules

A total of 841 high risk nodules were detected in all subjects, for a detection rate of 4.4%. Analysis showed that the detection rates of the pre- and postepidemic groups were not significantly different. Further analysis of each age and sex group showed that the difference was not statistically significant. Further details are shown in **Tables 1** and **3**.

**Table 3:** Comparison of the detection rates of high risk nodules at different ages between the preepidemic and postepidemic groups.

	Preepidemic group		Postepidemic group	χ2	χ2	P
	[n]	High risk nodules [n (%)]	[n]	High risk nodules [n (%)]		
Sex [n (%)]						
Male (n=11757)	5588	250 (4.5)	6169	275 (4.5)	0.002	0.966
Female (n=7149)	3236	153 (4.7)	3913	163 (4.2)	1.326	0.249
Age (years) [n (%)]						
≤40 (n=5843)	2506	112 (4.5)	3337	120 (3.6)	2.862	0.091
41~50 (n=5063)	2450	90 (3.7)	2613	108 (4.1)	0.711	0.399
51~60 (n=5685)	2771	151 (5.4)	1914	148 (5.1)	0.391	0.532
≥61 (n=2315)	1097	50 (4.6)	1218	62 (5.1)	0.355	0.551

## Discussion

COVID-19 is transmitted mainly through the respiratory tract, with the lungs being one of the main organs affected (11). On the other hand, lung cancer is known worldwide for its high morbidity and mortality and is the number one killer of malignant tumors. Fortunately, early screening of specific populations via low-dose spiral CT (LDCT) technology can significantly reduce the mortality rate of patients with lung cancer, and this effective method has been recommended by professional guidelines in several countries (12). However, the emergence of COVID-19 has introduced new challenges to the medical field. The CT imaging characteristics of some patients with COVID-19 are similar to those of early lung cancer patients, who present with ground-glass or solid nodules, which undoubtedly increases the difficulty of their differential diagnosis and treatment. Studies have shown that the late stage of COVID-19 may lead to an increase in the number of pulmonary nodules (13). However, it is unclear whether COVID-19 leads to increased numbers of lung nodules and lung cancer. This field needs to be studied in depth to understand the long-term effects of COVID-19 on lung health more accurately.

This study showed a higher detection rate of pulmonary nodules in the post-COVID-19 epidemic group compared to the pre-epidemic group, suggesting an increase in solid pulmonary nodules post-COVID-19. Our study also revealed that the increased nodules were mainly solid, and the increased pulmonary nodules were mainly concentrated in subjects over 51 years of age. A meta-analysis study found that the detection rate of pulmonary nodules and fibrosis increased one year after the post-COVID-19 period, which is consistent with our results (14). Wu et al (15). recently reported that in a study of 83 COVID-19 patients, residual ground-glass opacity (GGO) was still detectable in 24% of the cases. Studies have shown that in the early stage of COVID-19, lesions are mainly located in the lung periphery or under the pleural area, and ground-glass nodules are more common, which is related to the formation of transparent membranes in the alveolar wall (16). In the progressive stage of the disease, the distribution of lesions becomes wider, and ground-glass nodules may coexist with consolidation opacities and linear opacities and may be accompanied by lobular septal thickening. The pathologic mechanism is related to the early involvement of the alveolar wall by the novel coronavirus (17). In severe patients, diffuse lesions were usually seen in multiple lobes and segments of both lungs. These were mainly consolidations associated with ground-glass nodules and linear opacities. During the remission period, the lesions in both lungs gradually resolved and were relieved. CT showed that the ground glass lesions decreased in number and size. However, some of the lesions did not resolve and remained as solid nodules, which was associated with age and immune function (18, 19), Consistent with our findings. A study of 1953 patients with COVID-19 showed elevated levels of IL-6, IL-8, TNF-α and IL-1B. They showed that the novel coronavirus can induce an activated immune response, often referred to as “cytokine storming” (20). Another study showed that the pathogenicity of the COVID-19 cytokine storm was associated with increased numbers of Th 17 and highly proinflammatory CCR6+ CD4+ T cells in COVID-19 patients (21). These mechanisms may help to explain how COVID-19 causes pulmonary nodules to form. These findings also help to explain why pulmonary nodules tended to be detected in older participants. Therefore, we believe that the increased number of pulmonary nodules during the COVID-19 pandemic is mainly due to solid pulmonary nodules and mainly affects older, immunocompromised individuals.

Our study showed no increase in high risk nodules in the pre- and postepidemic groups. Consistent with the results of our study, we did not find any relevant reports on lung cancer caused by COVID-19 through a literature search. However, lung cancer is a risk factor for COVID-19-associated pneumonia in some studies. IL-6 levels are significantly increased in lung cancer patients with COVID-19, and these factors induce a cytokine storm that leads to acute respiratory distress syndrome. This leads to a shorter median survival time in COVID-19 patients with lung cancer (22).

Before the COVID-19 epidemic, the Chinese consensus recommendation for screening for pulmonary nodules was to perform chest CT in people aged 50-74 years (23). However, after the COVID-19 epidemic, our study showed that the detection rate of lung nodules increased in people older than 40 years (24, 25). The Chinese COVID-19 prevention guidelines use chest CT as one of the indicators for the diagnosis of COVID-19 infection, which also leads to more young people being willing to undergo chest CT examination (26, 27). These factors may have led to significantly higher rates of chest CT screening among people younger than 40 years after the COVID-19 outbreak than among those younger than 40 years before the outbreak. The relatively young demographics of the study cohort (only about 12% aged ≥61 years) reflect those of routine health screening participants in China. Our study included individuals under 40 years of age, reflecting the actual age distribution of participants in routine health screening in China. Although this group has a lower risk of lung cancer, their inclusion provides an important real-world context for our research. Although younger individuals have a lower inherent risk of lung cancer, their inclusion underscores the real-world difficulty of differentiating COVID-19-related inflammatory nodules from potential malignancies in asymptomatic individuals. This age distribution also likely contributes to the lack of a significant post-pandemic increase in high-risk nodules, as younger individuals are less susceptible to aggressive cancers (28). It’s worth noting that the inclusion of individuals below the age threshold recommended by most international lung cancer screening guidelines (29) (for instance, lung screening is only recommended for those > 50 years in the United States, Canada, most of Europe and South America).may limit the generalizability of our findings to populations with stricter eligibility criteria. Furthermore, the dominance of middle-aged and younger participants (88% <60 years) in our cohort may attenuate the observed association between COVID-19 and lung cancer risk, as age remains a key determinant of malignancy.

The results of this study showed that pulmonary fibrosis was detected at a higher rate in the post-epidemic group compared to the pre-epidemic group. The vast majority of survivors of severe COVID-19 have multiple sequelae. These include pulmonary, neurological, psychological, and cardiovascular complications (30). The lung is the main organ damaged by the novel coronavirus. The major long-term complication of COVID-19 is pulmonary fibrosis (31). A study of chest CT scans of patients recovering from COVID-19 infection showed that 38% of patients had persistent interstitial lung abnormalities, mainly manifested as ground-glass opacities (GGOs), spinal cord consolidation, and physiological damage. Some lesions gradually progressed to pulmonary fibrosis, which persisted during follow-up (32). Lung fibrosis may be a result of physiological injury (33). Research has shown that physiological injury to the lung leads to abnormal repair and fibrosis of lung tissue through the activation of multiple cells and signaling pathways (34).

Our study has several limitations. First, this is an observational study and requires further prospective research to confirm, but this opportunity may not be available in the future. Second, this study’s data came from examination centers. According to China’s national medical insurance policy, the examination fees of medical examination centers are not covered by medical insurance reimbursement. Therefore, there may be a certain degree of economic selection bias, as the people who participated in the study were mainly those who voluntarily underwent medical examinations and could pay the fees themselves. This selection bias may further affect the representativeness of the research results. Third, although this is a multicenter study, we only included data from three hospitals. This study was conducted on the basis of three hospitals in the Chengdu area, and the results may be affected by geographical and institutional limitations. The three hospitals involved in the study are all located in the southern part of the city of Chengdu, which is a relatively concentrated geographical area. Urbanization in Chengdu is relatively complete, and the urban-rural gap in the region is small. However, this concentration may still limit the broad applicability of the research results. Fourth, the main results of this study were evaluated by CT scans, and some of the results were evaluated by physicians without pathological support. While our study highlights the changes in the detection rates of lung nodules and high-risk nodules before and after the COVID-19 pandemic, the actual incidence of lung cancer in this cohort requires longer follow-up. Future studies will include longitudinal data to evaluate the progression of high-risk nodules and calculate cancer incidence rates.

## Conclusion

The COVID-19 pandemic resulted in more young people aged <40 years undergoing chest CT. With the increasing incidence of COVID-19, the detection rates of lung nodules and pulmonary fibrosis lesions increased, mainly due to an increase in the number of solid lung nodules. These increases were mainly concentrated in people over 51 years of age. The COVID-19 pandemic did not increase the detection rate of high risk nodules.

## Data Availability

The datasets generated and/or analyzed during the current study are contained within the Office of Thoracic Surgery, First People’s Hospital of Shuangliu District but are not publicly available because confidentiality, security and ownership matters. They may be available from the corresponding author upon reasonable request.
